# Bisphenol A and Infant Neonatal Neurobehavior: Sathyanarayana et al. Respond

**DOI:** 10.1289/ehp.1104429R

**Published:** 2012-03-01

**Authors:** Sheela Sathyanarayana, Joe Braun, Kimberly Yolton, Stacey Liddy, Bruce Lanphear

**Affiliations:** Department of Pediatrics, University of Washington, Seattle, Washington, E-mail: sheela.sathyanarayana@seattlechildrens.org; Department of Environmental Health, Harvard School of Public Health, Boston, Massachusetts; Department of Pediatrics, Division of General and Community Pediatrics, Cincinnati Children’s Hospital Medical Center, Cincinnati, Ohio; Children’s Hospital and Simon Fraser University, Vancouver, British Coulmbia, Canada

In their letter, Haighton et al. recommend that we review Bradford Hill guidelines for establishing causality. As noted in our article (Sathyanarayana et al. 2011), we did not try to establish cause and effect with this single case study and cautioned against doing so. In medical research, new syndromes or toxicants have often been identified by the report of unusual cases or exposures, even though it is not always apparent with the initial case report. Thus, we believe that it is important to highlight this and other unusual cases to identify potential causative agents of neurobehavioral abnormalities in childhood. This case study does not stand in isolation; there is a growing body of animal and human literature documenting the neurodevelopmental impacts of bisphenol A (BPA) exposure (Braun et al. 2009; National Toxicology Program, Center for the Evaluation of Risks to Human Reproduction 2008; Yolton et al. 2011).

Haighton et al. state that glucuronidated BPA does not appear to be biologically active in mammalian systems; however, glucuronidated BPA can be deconjugated by the placenta or transferred across the placenta, where it can be deconjugated by other fetal tissues (Ginsberg and Rice 2009; Nishikawa et al. 2010). Therefore, a developing fetus can be exposed to a biologically active substance. Also, more recent literature has noted that BPA may be metabolized into other biologically active agents through oxidative cleavage to create the estrogenically active metabolite 4-methyl-2,4-bis(4-hydroxyphenyl)pent-1-ene (MBP), which is reported to be 500 times more potent than BPA *in vivo* (Okuda et al. 2010).

The authors were unclear about how “normal limits” for neurobehavioral assessment are defined. In our study (Sathyanarayana et al. 2011), we administered a wide range of standardized tests that include validated threshold values that can be used to identify children at risk for delayed development or clinically significant behavioral problems (Bayley 1993; Gioia et al. 2003; Wechsler 2002). The comparison of the case child to these values is valid because the distribution of scores for children in our study are comparable to those in nationally representative population-based samples used to validate these instruments. This is how these tools are used clinically to identify children who may have neurobehavioral abnormalities.

Finally, Haighton et al. state that other etiologies could be responsible for the abnormal exam at 1 month of age. We stated this exact same point very clearly in our case report (Sathyanarayana et al. 2011). Although these other etiologies may be important in infant and child neurodevelopment, they would be confounders only if they are associated with both BPA exposure and neurodevelopment (Hernan et al. 2002).
